# Overcoming the Elusiveness of Neurosarcoidosis: Learning from Five Complex Cases

**DOI:** 10.3390/neurolint13020013

**Published:** 2021-03-25

**Authors:** Parissa Feizi, Medha Tandon, Erum Khan, Roshan Subedi, Apoorv Prasad, Anisa Chowdhary, Shitiz Sriwastava

**Affiliations:** 1 Department of Neuroradiology, Rockefeller Neuroscience Institute, West Virginia University, Morgantown, WV 26505, USA; pfeizi@hsc.wvu.edu; 2 Safdarjung Hospital, New Delhi 110029, India; medhatandon22@gmail.com; 3 B.J. Medical College and Civil Hospital, Ahmedabad 380016, India; erum2006@gmail.com; 4 Institute of Medicine, Kathmandu P.O. Box 1524, Nepal; roshansubedi123@gmail.com; 5 Department of Neurology, Berkeley Medical Center, West Virginia University, Morgantown, WV 26506, USA; apoorvprasad@gmail.com; 6 Institute of Nuclear Medicine and Allied Sciences, New Delhi 110054, India; anisaanila@gmail.com; 7 Department of Neurology, Rockefeller Neuroscience Institute, West Virginia University, Morgantown, WV 26505, USA; 8 Department of Neurology, Wayne State University, Detroit, MI 48201, USA; 9 West Virginia Clinical and Translational Science Institute, Morgantown, WV 26505, USA

**Keywords:** neurosarcoidosis, CSF in neurosarcoidosis, MRI in neurosarcoidosis, orbital sarcoidosis, ring enhancing lesion, primary CNS sarcoidosis

## Abstract

The involvement of the central nervous system in sarcoidosis can manifest with a variety of neurological symptoms, and most of them can be nonspecific. The diagnosis of neurosarcoidosis (NS) can therefore be very challenging without a tissue biopsy. Both computed tomography (CT) and magnetic resonance imaging (MRI) are important imaging modalities in the diagnosis of NS, and MRI is the modality of choice due to its superior soft-tissue contrast resolution. We present a case series of NS with interesting neuroimaging features, complex neurological presentations, and clinical courses. We identify five cases presenting with clinically isolated neurosarcoidosis (CINS) without any other signs or symptoms of systemic disease which were diagnosed as NS on biopsy. In the first case, we describe a patient with an intramedullary cervical spinal cord lesion. In the second case we describe a patient presenting with inflammatory changes and enhancement in the orbit. The third case demonstrates a lesion with calcification around the region of the foramen of Monro. The fourth case shows multiple ring-enhancing lesions. Lastly, the fifth case exhibits unusual findings with both optic neuritis and a cerebellar nodule. We aim to describe the complicated clinical course with neurological workup, neuro-imaging, and eventual diagnosis and treatment of these challenging cases to highlight the variable presentations of NS. This case series will remind clinicians that NS should always be in the differential diagnosis when a patient presents with nonspecific neurological symptoms with unusual neuroimaging findings.

## 1. Introduction

Sarcoidosis is a multi-systemic idiopathic granulomatous disorder without any definite diagnostic parameters associated with its pathophysiology, posing a clinical challenge from its onset [1,2,3,4,5,6]. Virtually no organ system has been left unaffected in sarcoid literature, with the most common involvement being the pulmonary system (up to 90% of patients) [7,8]. The unique subtype of sarcoid limited to the central and peripheral nervous system has been identified in approximately 5–15% of patients is known as NS [2,8,9]. NS poses an even greater diagnostic challenge because of isolated organ system involvement which is not easily amenable for tissue biopsy [1,7]. NS patients can present with varying signs and symptoms inclusive but not limited to myelopathy, meningitis, hydrocephalus, seizures, headache and neuropathies [2,3,4,9,10].

The gold standard in diagnosis and differentiation of NS from other diseases like multiple sclerosis (MS) remains tissue biopsy, which is not always feasible considering the requirement of both a biopsy-appropriate site of lesion and expertise [1,11,12,13,14]. However, neuroimaging findings when complemented with CSF findings may have the potential to diagnose NS early when tissue biopsy is unavailable or not feasible [1,4,15].

Literature in the past has described that the diagnosis of neurosarcoidosis can be challenging even after testing for spinal fluid or serum ACE levels and nonspecific imaging findings [12]. A strong degree of clinical suspicion and a combination of neuro imaging and histopathology would greatly aid in a timely diagnosis, leading to earlier management. We aim to provide further insights into this elusive diagnosis through our case series of five patients.

## 2. Design/Methods

Five cases of neurosarcoidosis were identified at the authors’ institution and associated hospitals. Data analysis included age, sex, symptoms, risk factors, treatment, and imaging findings.

## 3. Results

### 3.1. Case 1

A 27-year-old Caucasian female presented with right facial droop and numbness, otalgia, metallic taste, left sided weakness, nausea and dizziness for one week. She was started on oral antibiotics and steroids for suspected ear infection and Bell’s palsy.

Cranial nerve exam demonstrated lower motor neuron right facial nerve palsy. Her facial nerve dysfunction was grade 5 (severe dysfunction) and physical exam also revealed decreased facial sensation to light touch on the right side. Strength was 3/5 on the left arm and left leg in all muscle groups and 5/5 on the right side with brisk reflexes throughout and up going plantar response on the left side.

Due to clinical concern for infectious etiologies including lyme disease or viral infection, the patient was started on empiric treatment with 100 mg doxycycline, 500 mg valacyclovir twice a day and continued 60 mg oral prednisone daily. The serum autoimmune and infectious work up was unremarkable including antinuclear antibodies (ANA), neutrophil cytoplasmic antibodies (ANCA), ENA screen SS-A/Ro and SS-B/La antibodies, Anti-Smith antibodies, RNP antibodies, Anti-Scl-70 antibodies, Anti-double stranded DNA antibodies, Anti-chromatin antibodies, Anti-centromere antibodies, and antimitochondrial antibodies.

**Figure 1:**
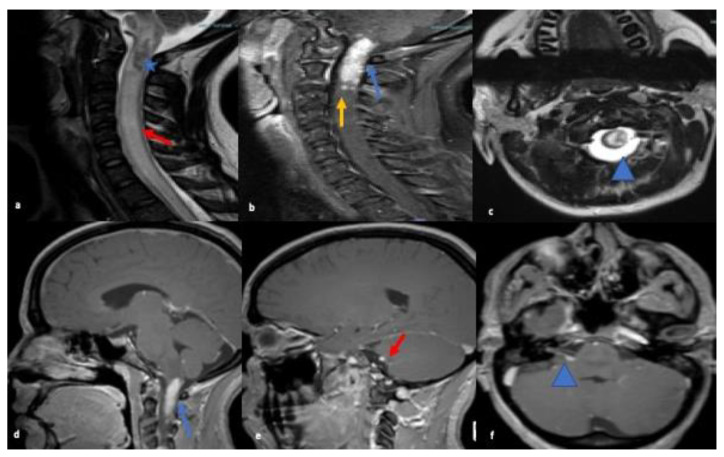
MRI sagittal T2 images (**a**) of the cervical spine reveal ill-defined long segment signal alteration with prominent cord expansion (red arrow) and ill-defined heterogenous lesion in cervicomedullary junction of the cord (blue asterisk); and (**b**) T1 post contrast image shows cord enhancement (blue arrow) and multiple separated subtle foci of enhancement were noted at C3 (yellow arrow). Axial T2-weighted (**c**) reveals corresponding hyperintense cord signal (blue arrowhead). MRI Brain sagittal and axial T1 post contrast images highlights homogenous enhancement at cervicomedullary junction (blue arrow; (**d**)), and enhancement along CN VII and VIII nerve complex (red arrow; (**e**)) & (blue arrowhead; (**f**)).

MRI brain showed abnormal enhancement within the right internal auditory canal with extension to the right cerebellopontine angle to the root of the VII and VIII cranial nerve complexes. MRI C-spine showed intramedullary enhancing lesions extending throughout the cervical spinal cord spanning C1 and C2. Multiple subtle foci of enhancement were also noted at C3 and within the lateral aspect of the medulla (refer to Figure 1). CT chest/abdomen/pelvis were without evidence of malignancy or systemic sarcoid.

A lumbar puncture was performed, and relevant CSF findings included elevation of CSF protein of 77 mg/dL and cell count of 10 cells/mm^3^ with predominant lymphocytes. CSF kappa free light chain was normal. Biopsy of the cervical intramedullary lesion indicated granulomatous inflammation and no neoplastic or primary demyelinating process. Based on these findings, the patient was diagnosed with NS and was started on high dose (1 gm) IV methylprednisolone daily for five days. Patient showed some improvement in muscle strength on day 4 of treatment. She was discharged to inpatient rehab with continuation of oral steroids (refer Table 1).

### 3.2. Case 2

A 51-year-old Non-Hispanic female with a past history of surgically resected fibroadenoma, presented with three weeks of gradually progressive left eye swelling, pain with extraocular movements and diplopia with decreased visual acuity (20/200 on left and 20/20 on the right). CT scan of the brain in the emergency department showed findings concerning for orbital cellulitis with extensive phlegmonous changes of the retrobulbar fat and a retro-orbital fluid collection suspicious for an abscess. Mild proptosis of the left globe was also noted.

**Figure 2:**
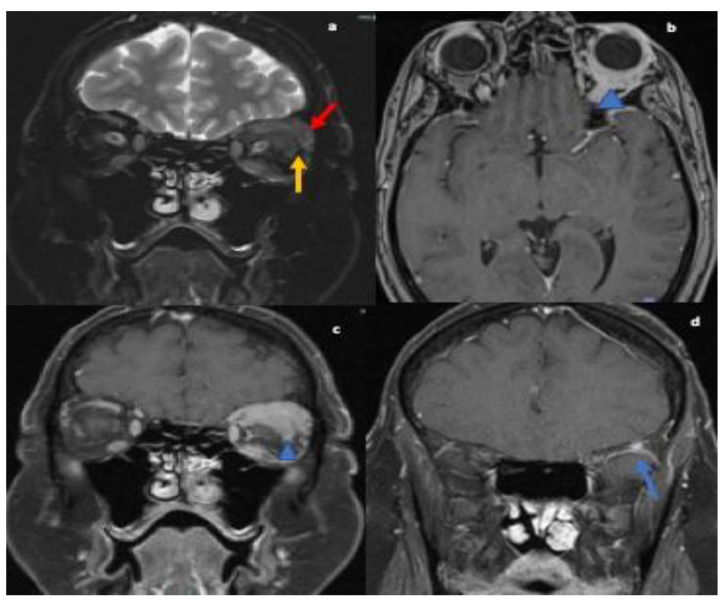
MRI orbit T2 fat-suppressed coronal images reveals nodular thickening of the left lacrimal gland (red arrow; (**a**)), superior and lateral recti muscles (yellow arrow; (**a**)). T1-weighted post contrast axial (**b**) and coronal images (**c**) showing corresponding enhancement within muscles, lacrimal gland and in intraconal orbits surrounding the globe (blue arrowhead).T1 weighted post contrast coronal image (**d**) reveals enhancing nodule in the greater wing of left sphenoid concerning for osseous involvement (blue arrow).

The serum autoimmune and infectious work up were unremarkable. MRI orbits showed soft tissue thickening and enhancement in the left orbit with enlarged extraocular muscles, enlarged lacrimal gland and osseous involvement of the sphenoid bone (refer Figure 2). Differential at that time included cellulitis and lymphoproliferative disorder. She was started on oral antibiotics followed by left orbitotomy and excision of the mass. Biopsy of the mass revealed granulomas and the patient was diagnosed with orbital sarcoidosis. CT chest/abdomen/pelvis were without evidence of malignancy or systemic sarcoid. No lumbar puncture was performed as diagnosis was confirmed on biopsy.

She was started on oral prednisolone 10 mg daily, methotrexate 10 mg weekly; later increased to 15 mg weekly maintenance dosage. On her follow up visit, disease activity improved. The diplopia had resolved and the patient’s vision returned to baseline (20/20) bilaterally. She was continued on her medications with a reduction in methotrexate dose to 12.5 mg weekly and is being gradually tapered off of steroids (refer Table 1).

### 3.3. Case 3

**Figure 3:**
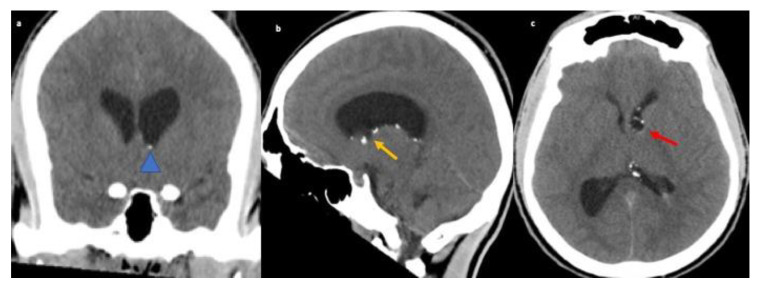
CT Head non-contrast coronal (**a**) reveals small punctuate foci of calcification along the left lateral ventricle (blue arrow head). Additional CT head sagittal and axial (**b**,**c**) reveals 9 mm × 6 mm fluid density lesion in the frontal horn of left lateral ventricle with peripheral calcification near foramen of Monro (yellow and red arrow) leading to obstructive supratentorial hydrocephalus. Additional small punctuate foci of calcification along the left lateral ventricle.

A 26-year-old African-American male with no significant medical history presented to the ED with decreased level of consciousness complaining of severe headache, nausea, vomiting and bowel and bladder incontinence for one day. The patient underwent emergent CT Head and CT angiogram to rule out subarachnoid hemorrhage. CT Head showed ventriculomegaly and diffuse sulcal effacement concerning for edema. A 9 × 6 mm fluid density lesion in the frontal horn of left lateral ventricle adjacent to the foramen of Monro with peripheral calcification was identified (yellow and red arrow), leading to obstructive supratentorial hydrocephalus (blue arrow head and yellow arrow). Additional scattered hyperdensities were also seen along the left lateral ventricular wall (refer Figure 3).

The patient underwent external ventricular drain (EVD) placement with opening pressure of 22 cm of water and underwent surgery for removal of the lesion at the foramen of Monro. Pathology report from the biopsy of the lesion revealed choroid plexus with predominantly non-caseating granulomatous inflammation and intraventricular calcifications. A gradual improvement in mentation was noted following the procedure. He also showed improvement in headache.

Laboratory workup was unremarkable for infectious or other autoimmune markers. CSF studies were remarkable only for elevation in CSF protein (67 mg/dL). The patient was then started on prednisone after post-biopsy confirmation of neurosarcoidosis, and was transferred to inpatient rehab. On follow up evaluation in clinics after two months, the patient’s symptoms had resolved. He was advised to continue prednisone 10 mg daily as the biopsy findings were concerning for NS.

No MRI images of the foramen of Monro lesion in Figure 3 are not available. We believe that the CT head images in Figure 3 with the support of the final pathology of the lesion suffices for the diagnosis of NS (refer Table 1).

### 3.4. Case 4

**Figure 4:**
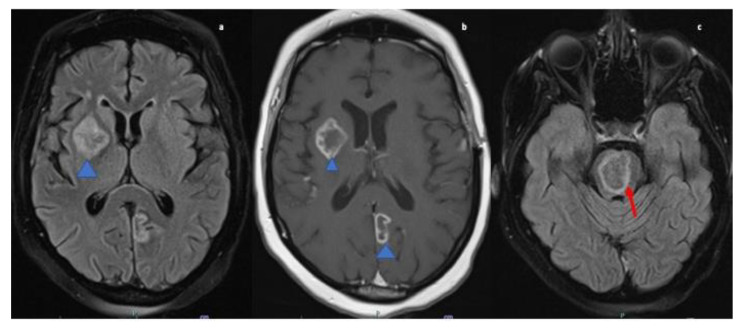
MRI Brain axial fluid-attenuated inversion recovery (FLAIR; (**a**)) & T1-weighted post contrast image (**b**) reveals hyperintense lesion in right gangliocapuslar region (blue arrowhead; (**a**)) with corresponding heterogenous peripheral ring enhancement, in addition to enhancement in the left paramedian occipital lobe (blue arrow head; (**b**)). FLAIR image (**c**) reveals circumscribed intra-axial parenchymal lesion in the pons (red arrow).

A 47-year-old African American female with past medical history of hypertension was transferred from an outside hospital after presenting with left sided upper and lower extremity weakness., MRI brain was performed which demonstrated multiple ring-enhancing lesions on post contrast T1 images, the largest within the right basal ganglia and the pons. There was also a smaller lesion in the left medial occipital lobe (refer Figure 4. The findings were concerning for possible demyelinating disease such as tumefactive multiple sclerosis, metastases or infection. A CT chest, abdomen, and pelvis revealed uterine masses likely representing uterine fibroids but otherwise no evidence of malignancy. Subsequently MRI of the pelvis was performed for further work up of the uterine masses which showed the large heterogeneous uterus was most likely due to degenerative fibroids and no suspicious uterine lesion was seen. Lumbar puncture was not performed due to concern for increased ICP. TTE was performed to rule out infective endocarditis and showed no evidence of valvular vegetations. Autoimmune and infectious serum panel were all negative.

A brain biopsy was performed and final pathology showed necrotizing granulomas with associated calcifications consistent with NS. Biopsy stains for acid-fast and fungal (methenamine silver and PAS) organisms, with appropriate positive controls, were performed and were negative. A Congo red stain, with an appropriate positive control, was also performed and was negative. The patient was started on methylprednisolone 1 g daily for a total of five days. At the time of discharge the patient was switched to prednisone 60 mg daily for a total of 30 days and advised to follow up in the neurology clinic in four weeks after discharge. The patient reported an improvement in the left sided weakness on his follow-up visit. In addition to prednisone 60 mg daily, the patient was prescribed omeprazole 20 mg daily and advised to continue with home medications (refer Table 1).

### 3.5. Case 5

A 50-year-old Caucasian male with past history of hyperlipidemia and polycythemia vera presented to an outside hospital with chief complaint of bilateral retro-orbital pain and progressive vision loss over the course of two months. The patient denied weakness, numbness, tingling, seizures, neck pain, back pain, or history of incontinence. On examination, vision was 20/70 in the right eye. The left eye was reported to have complete visual loss.

**Figure 5:**
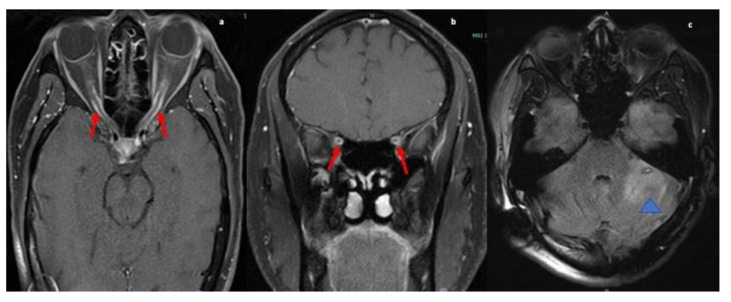
MRI orbit axial T1-weighted fat-suppressed post contrast image (**a**) and coronal (**b**) images reveal enhancement of bilateral optic nerve sheaths (red arrow). MRI Brain axial fluid-attenuated inversion recovery (FLAIR) (**c**) image reveals ill-defined patchy parenchymal hyperintensity in the left cerebellar hemisphere extending to the cerebellar peduncle (blue arrowhead).

MRI brain and orbits revealed an enhancing left cerebellar nodule with surrounding abnormal hyperintense signal on FLAIR extending to the cerebellar peduncle. Mass-like enhancement of the folia surrounding the nodule was also noted. MRI orbits showed bilateral smooth optic nerve sheath enhancement without abnormal signal within the optic nerves. There were also imaging findings concerning for papilledema with flattening of the posterior globes (refer Figure 5) and T2 images (not shown) also demonstrated intraocular protrusion of the optic nerve heads. These findings were most consistent with inflammatory processes such as sarcoid or possible leptomeningeal metastatic disease.

A lumbar puncture was performed, and a VP shunt was placed. CSF analysis revealed CSF protein of >400 mg/dL, elevated opening pressure, CSF ACE mildly elevated at 3.7 U/L (normal up to 2.5), 12 WBC predominant lymphocytic 90%, and negative meningitis panel. Serum ACE was normal. CT chest/abdomen/pelvis were without evidence of malignancy or systemic sarcoid.

The patient received left optic nerve sheath fenestration in an effort to decrease the swelling of the optic nerve. Biopsy of the left optic nerve sheath demonstrated findings concerning for NS. The patient was started on one dose of cyclophosphamide with Mesna and discharged with oral tapering prednisone starting at 60 mg dose, with a follow-up scheduled in neurology and ophthalmology clinics. Following the treatment, the patient continued to have lack of light perception in left eye but the visual acuity improved within the right eye to 20/40. Recommendation was to follow-up with the neurology outpatient clinic (refer Table 1)

## 4. Discussion

Sarcoidosis, a chronic granulomatous disorder, is a product of genetic predisposition and pathological mechanisms initiated by T-lymphocytes to an unidentified antigen [16]. Described as a “mimic” of various nervous system manifestations, neurosarcoidosis is a diagnosis of exclusion [1]. However, a higher degree of clinical suspicion and neuro-radiological correlation can lead to early diagnosis, treatment and a better outcome.

NS can affect the neurological system in various ways. One of these is cranial nerve involvement. Any of the cranial nerves can be involved in NS [11] but the most frequently reported is cranial nerve VII [1]. The first case in our series presented with lower motor neuron VII nerve palsy and VIII nerve involvement leading to a misdiagnosis of Bell’s palsy and ear infection. Multiple cranial nerve involvement, lesion within the brainstem and intramedullary lesions within the upper cervical spinal cord were seen on MRI were present in this case of NS. Despite treatment with steroids, the patient did not initially show improvement, complicating the case even further. The second case, initially diagnosed as orbital cellulitis, also demonstrated multiple cranial nerve involvement with ophthalmoplegia secondary to infiltration of the extraocular muscles due to granulomatous masses. The patient experienced loss of vision in the left eye due to surrounding inflammatory changes and mass effect. A subsequent biopsy confirmed the diagnosis of orbital sarcoidosis. Hence, cranial nerve involvement with other CNS lesions should raise suspicion for NS and treatment with steroids may not show a significant response initially.

Spinal cord predominant NS may involve leptomeningeal, pachymeningeal, and intramedullary regions, mimicking other myelopathies presenting with sensory, motor, and autonomic dysfunction [17,18,19]. Cervical and thoracic segment involvement is more commonly reported than lumbosacral involvement, similar to our case [19,20,21]. Junger et al. organized MRI manifestations ranging from Stage 1, indicating early inflammation to Stage 4, indicating progressive spinal cord atrophy [20].

Our first case presented with diffuse abnormal T2 signal throughout the cervical cord and intramedullary cervical cord enhancement with no cord atrophy consistent with stage 1 disease and involved more than three vertebral segments. In the past, authors have recommended using the length of spinal cord signal abnormality to aid in etiology of the lesions [14,17,18,19,20]. NS most commonly presents as multisegmental disease spanning multiple levels in the cord, uncommon in multiple sclerosis where plaques are usually shorter than two vertebral body lengths. However, longitudinally extensive spinal cord lesions (>3 vertebral segments on MRI examination) with acute myelitis are a characteristic feature of neuromyelitis optica spectrum disorders (NMOSD), which closely mimics NS, often leading to misdiagnosis [22,23]. Meningeal enhancement or persistent enhancement (more than a few weeks) of parenchymal lesions are much more suggestive of sarcoidosis than NMO [24]. Also, testing for the NMO/AQ-4 antibody is helpful in this setting, and was negative in our patient.

The second case, initially diagnosed as orbital cellulitis, had second cranial nerve involvement with marked loss of vision in the left eye. There was also infiltration of extraocular muscles with pain upon eye movements and ophthalmoplegia due to granulomatous mass. Orbital sarcoid manifestations include thickening and enhancement of the intra-orbital and intracranial optic nerve and chiasm, optic atrophy and orbital masses. These are often mistaken as pseudotumor, glioma, meningioma of the optic nerve sheath, MS, or infectious etiology such as syphilis, lymphogranuloma venereum, leprosy, tularemia, torulosis, histoplasmosis, blastomycosis, and coccidioidomycosis [25,26,27,28,29]. Fast spin-echo fat suppressed axial and coronal T2, and T1 axial and coronal fat-suppressed contrast-enhanced MRI images are considered to be the standard protocol to investigate for intra-orbital or optic nerve disorders [30].

Hydrocephalus is present in 5–12% of patients with NS [31,32]. Obstructive lesions with leptomeningeal involvement can result in hydrocephalus in patients with NS with neuro-infectious diseases forming its prime differential diagnosis [11,33]. Case three presented with extreme headache and altered consciousness as a consequence of increased intracranial pressure with granulomatous mass obstructing the ventricular system. The biopsy confirmed NS. With the absence of leptomeningeal or dural involvement, this unique case posed a challenge in ruling out the differential of infectious disease which includes TB, fungal infections such as Blastomycosis and Histoplasmosis, Spirochetal infection, and several other unusual microbes like Bartonella [11]. Moreover, a linear or nodular ependymal enhancement like this case can also be suggestive of tumoral, cystic, vascular, and inflammatory pathology. Thus, calcified choroidal lesions in the ventricular system may be the only presentations of NS without parenchymal or spinal involvement [32,33].

Non-specific MRI findings such as periventricular white matter lesions, meningeal enhancement, brain parenchymal lesions, dural mass lesions, and spinal cord involvement are commonly reported in NS [1,34,35]. However, ring-enhancing lesions, though reported rarely, may be the only presentation of NS [36,37]. Case four presented with increased intracranial pressure due to cerebral vasogenic edema and multiple ring-enhancing lesions adding neoplastic lesions, demyelinating diseases, contusions, and Wegner’s granulomatosis to the differential list. Such a case poses unique challenges in diagnosis as many of the differentials cannot be excluded immediately because of inability to perform LP in the presence of cerebral edema. However, whole-body imaging narrowed down the diagnosis of NS and it was confirmed by biopsy.

The presenting symptoms of case five were retro-orbital pain, blurring of vision progressively leading to complete diminution and fundoscopic examination revealing papilledema consistent with increased ICP. However, cerebellar and cervical leptomeningeal enhancement were also discovered on MRI, and the patient exhibited no symptoms from these lesions. Hence, it appears that in some cases of NS the imaging findings may not correlate entirely with the clinical presentation of the disease.

Neuroimaging findings in sarcoidosis have been described in the literature with some classical signs including “Trident sign”. The trident sign as described in literature by Zalewski et.al. and Jolliffe EA et.al, is the MRI appearance on the axial post contrast of the spine with central cord enhancement along with subpial enhancement resembling a trident pattern strongly suggestive of NS. It has a significant diagnostic yield to distinguish NS from other causes of myelitis such as neuromyelitis optica (NMO) and other longitudinal extensive cord lesions [23,38].

Intraparenchymal imaging findings of NS include white matter lesions along the periventricular region indistinguishable from multiple sclerosis (MS) and vascular disorders. The enhancing intraparenchymal lesions are often seen with corresponding leptomeningeal or pachymeningeal disease. In about 30–40% of NS cases, leptomeningeal involvement presents with diffuse or nodular enhancement and thickening of the leptomeninges. Common regions involved are suprasellar area as well as basilar meninges. Enhancement may spread along the perivascular spaces and lead to intraparenchymal lesions. Though any cranial nerve can be involved in NS, the most common cranial nerve abnormality seen on neuroimaging modalities is optic nerve (CN II), while the commonest cranial nerve deficit reported is CN VII [16,39].

Although biopsy of the brain or spinal cord tissue was performed for the definitive diagnosis in our series, it may not always be feasible. Furthermore, an attempt to biopsy from difficult to access areas may increase morbidity and mortality [21,40]. Our series intends to make clinicians aware of the atypical presentations and neuroimaging findings that might point towards the possibility of NS and help formulate a management plan, even in the absence of tissue diagnosis.

Accurate and rapid diagnosis of NS persists to be a significant challenge in the field of neuroimmunology. Current recommendations greatly rely on MRI findings which include enhancing parenchymal lesions, T2/FLAIR periventricular white matter lesions, meningeal enhancement, hydrocephalus, cranial nerve involvement, dural masses and spinal cord involvement as well as increased systemic uptake on FDG-PET imaging. These features contribute to a striking number of differentials including infections, primary neoplasms, metastases and demyelinating conditions. Granulomatous change on biopsy with negative cultures of the lesions were confirmatory of isolated NS. These cases add to the spectrum of clinical and radiological features bringing NS higher in the list of differential diagnosis allowing rapid diagnosis and timely intervention.

## 5. Conclusions

Patients with complex neurological presentations in the setting of challenging CSF analysis and atypical MRI findings should prompt the physician to consider primary CNS sarcoidosis in the differential. Consequently, understanding the broad spectrum of clinical presentations and diagnostic modalities for this particularly rare form of disease has been extremely challenging in the field of neurology. Tissue biopsy of the lesions may not always be a possibility in suspected case. Therefore, understanding the varying clinical presentations, MRI imaging findings and CSF findings is crucial to timely diagnosis. Given the vast spectrum of clinical presentations and the rarity of disease incidence in the community, it is required to identify and report atypical presentations of NS. Further, large cohort studies are needed to gauge the importance and relevance of neuroimaging studies in the early diagnosis and management of neurosarcoidosis.

## Figures and Tables

**Table 1:** Clinical demographics, characteristics and Serum, CSF MRI findings and outcomes of patients with Neurosarcoidosis.

Case	Age/ Gender	Neurological Presentation	Serum Autoimmune and Infectious Panel	CSF Findings	MRI/CT Imaging Findings	Treatment and Outcome
1	27 y/F	1-week history of facial droop, numbness on right side of face and left sided weakness	Unremarkable for ANA, ANCA panel, ENA screen SS-A/Ro and SS-B/La antibodies, Anti-Smith antibodies, RNP antibodies, Anti-Scl-70 antibodies, Anti-double stranded DNA antibodies, Anti-chromatin antibodies, Anti-centromere antibodies, and antimitochondrial antibodies. VDRL, Human immunodeficiency virus antibody and lyme serum antibody were all negative.	CSF showed elevated WBC 10/mm^3^, predominant lymphocytes, protein 77 mg/dL, CSF glucose 69 mg/dL, serum glucose 157 mg/dL AQ-4 antibody was negative. Oligoclonal bands absent. CSF ACE not elevated values 0.6 U/L (ref range 0.0 to 2.5)	MRI brain and cervical spine abnormal enhancement identified within the right internal auditory canal with extension to the right cerebellopontine angle to the root of the VII, VIII cranial nerve complex with hyper-enhancing non-hemorrhagic intramedullary cervical spinal cord spanning the C1 and C2. Multiple separated subtle foci of enhancement at the C3 and within the lateral aspect of the medulla.	Findings from extensive radiological investigations, increased suspicion of sarcoidosis, for which confirmatory biopsy of intradural intramedullary lesions was done. Patient showed some improvement in muscle strength on day 4 of treatment with iv methylprednisolone 1 gm daily for 5 days. She was, then discharged to inpatient rehab with continuing oral steroids.
2	51 y/F	3 weeks of gradually progressive left eye swelling and pain with extra-ocular movements and diplopia with decreased visual acuity.	Unremarkable including ANA, ANCA, ENA screen SS-A/Ro and SS-B/La antibodies, Anti-Smith antibodies, RNP antibodies, Anti-Scl-70 antibodies, Anti-ds-DNA antibodies, Anti-chromatin antibodies, Anti-centromere antibodies, and antimitochondrial antibodies, VDRL, HIV antibody and lyme serum antibody were negative.	CSF study was not performed	MRI orbits showed soft tissue thickening and enhancement with enlarged recti muscles, lacrimal gland and enhancing nodule in the greater wing of left sphenoid concerning for osseous involvement	After subsidence of acute inflammation, the patients were subjected to surgical excision of the mass. Its biopsy confirmed the diagnosis of sarcoidosis which was treated with steroids and showed improvement. Prednisolone 10 mg daily, methotrexate 15 mg weekly maintenance dosage.
3	23 y/M	1-day history of worsening headache with decreased level of consciousness. Also, bowel and bladder incontinence	All infectious and autoimmune disease lab panels were negative as other cases.	CSF WBC 4/mm^3^, protein 67 mg/dL, CSF glucose 56 mg/dL, serum glucose 101 mg/dL AQP-4 antibody was negative. Oligoclonal bands absent. CSF ACE not elevated values 0.7 U/L (ref range 0.0 to 2.5)	CT brain showed enlarged lateral ventricles and hyper-density around the ventricles with effacement of sulci likely from raised intracranial pressure secondary to obstructive hydrocephalus. Also noted to have calcification in anteroinferior aspect of third ventricle and a nodular mass-like lesion in the foramen of Monro.	Patient underwent an External Ventricular drain (EVD) placement. He was started on prednisone post-biopsy confirmation during admission. On follow up evaluation at 2 months after discharge, patient’s symptoms had resolved and was recommended to continue prednisone 10 mg daily.
4	47 y/F	1-week history of fall at home Referral from another facility due to concerning lesions in the brain Weakness on the left upper and lower extremity	All infectious and autoimmune disease lab panels were negative as other cases.	CSF study was not performed	Multiple peripheral enhancing lesions involving the brainstem and supratentorial brain region. Some of the lesions demonstrated surrounding vasogenic edema.	During admission, biopsy confirmed the diagnosis of sarcoidosis which was well managed by steroids. Methylprednisolone 1 g daily for a total of 5 days. The patient reported an improvement in the left sided weakness on his follow-up visit. In addition to prednisone 60 mg daily
5	50 y/M	8-weeks history of headache behind the eyes with bilateral and progressively worsening vision and blurriness persisting despite refractory correction.	All infectious and autoimmune disease lab panels were negative as other cases.	CSF WBC 12/mm^3^ predominant lymphocytes, protein >400 mg/dL, CSF glucose 92 mg/dL, serum glucose 188 mg/dL AQP-4 antibody was negative. Oligoclonal bands absent. CSF ACE elevated values 3.7 U/L (ref range0.0 to 2.5)	MRI orbit images reveal enhancement of bilateral optic nerve sheaths. MRI Brain image reveals ill-defined patchy parenchymal hyperintensity in the left cerebellar hemisphere extending to the cerebellar peduncle.	The patient received left optic nerve sheath fenestration in effort to decrease the swelling in the optic nerve. Biopsy of the left optic nerve sheath confirm the diagnosis of sarcoidosis Patient was started on 1 dose of cyclophosphamide with Mesna and discharged with oral tapering prednisone starting at 60 mg dose and follow up in neurology and ophthalmology clinic. Following the treatment, the patient continued to have no light perception in left eye but the visual acuity improved on right eye to 20/40, and recommendation was to follow-up with the neurology outpatient clinic.

M, Male; F, Female; CSF, Cerebrospinal Fluid; ACE, Angiotensin converting enzymes; MRI, Magnetic resonance imaging; VDRL, Venereal Disease Research Laboratory test; AQ-4, Aquaporin-4 antibody.

## Data Availability

No reported data.

## Abbreviations

MRI	Magnetic Resonance Imaging
CSF	Cerebrospinal fluid
EVD	External Ventricular drain
ACE	Angiotensin converting enzymes
CINS	Clinically isolated primary Neurosarcoidosis
LP	Lumbar Puncture
TTE	Transthoracic Echocardiography
ICP	Intracranial Pressure; CNS, Central Nervous System
NMO	Neuromyelitis Optica
MS	Multiple Sclerosis
CN	Cranial Nerve
